# Prevalence of G6PD deficiency and diagnostic accuracy of a G6PD point-of-care test among a population at risk of malaria in Myanmar

**DOI:** 10.1186/s12936-023-04559-6

**Published:** 2023-05-01

**Authors:** Than Htike Aung, Chayanut Suansomjit, Zaw Min Tun, Tin Maung Hlaing, Jaranit Kaewkungwal, Liwang Cui, Jetsumon Sattabongkot, Wanlapa Roobsoong

**Affiliations:** 1 Mahidol Vivax Research Unit, Faculty of Tropical Medicine, Mahidol University, Bangkok, Thailand; 2 Defence Services Medical Academy, Yangon, Myanmar; 3 Defence Services Medical Research Centre, Nay Pyi Taw, Myanmar; 4 Department of Tropical Hygiene, Faculty of Tropical Medicine, Mahidol University, Bangkok, Thailand; 5 Division of Infectious Diseases and International Medicine, Department of Internal Medicine, Morsani College of Medicine, University of South Florida, Florida, USA

**Keywords:** G6PD deficiency, G6PD variant, Diagnosis, Prevalence, Malaria, Myanmar

## Abstract

**Background:**

Over the past decade, the incidence of malaria has steadily declined in Myanmar, with *Plasmodium vivax* becoming predominant. The resilience of *P. vivax* to malaria control is attributed to the parasite’s ability to form hypnozoites in the host’s liver, which can cause relapse. Primaquine is used to eliminate hypnozoites but can cause haemolysis in glucose-6-phosphate dehydrogenase (G6PD)-deficient individuals. It is thus necessary to estimate the frequency and variant types of G6PD deficiency in areas where primaquine will be widely used for *P. vivax* elimination.

**Methods:**

In this study, a descriptive cross-sectional survey was conducted to determine the prevalence of G6PD deficiency in a population residing in Nay Pyi Taw, Myanmar, using a standard spectrophotometric assay, a rapid diagnostic test (RDT), Biosensor, and by genotyping G6PD variants.

**Results:**

G6PD enzyme activity was determined from 772 leukocyte-depleted samples, with an adjusted male median G6PD activity value of 6.3 U/g haemoglobin. Using a cut-off value of 30% enzyme activity, the overall prevalence of G6PD deficiency was 10.8%. Genotyping of G6PD variants was performed for 536 samples, of which 131 contained mutations. The Mahidol variant comprised the majority, and males with the Mahidol variant showed lower G6PD enzyme activity. The G6PD Andalus variant, which has not been reported in Myanmar before, was also identified in this study.

**Conclusion:**

This study provides a G6PD enzyme activity reference value for the Myanmar population and further information on the prevalence and variants of G6PD deficiency among the Myanmar population; it also evaluates the feasibility of G6PD deficiency tests.

**Supplementary Information:**

The online version contains supplementary material available at 10.1186/s12936-023-04559-6.

## Background

The essential housekeeping enzyme, glucose-6-phosphate dehydrogenase (G6PD), plays an important role in the functioning of red blood cells (RBCs). It is located on the X chromosome with a high genetic diversity [1]. G6PD enzyme produces NADPH [a reduced form of nicotinamide adenine dinucleotide phosphate). Infections, such as hepatitis B, the consumption of some foods, such as fava beans, and the administration of some drugs, such as primaquine, can produce free radicals (reactive oxygen species (ROS)]. NADPH can compensate for these free radicals and protect RBCs from oxidative damage [2]. Thus, G6PD activity and the production of NADPH are vital for protecting cells from oxidative stress. While other cell types possess other enzymes producing NADPH, G6PD is the only enzyme in RBCs that produce NADPH. Therefore, RBCs cannot generate G6PD and G6PD deficiency in RBCs can lead to haemolysis [3]. Among the haematological diseases, G6PD deficiency is the most common disorder [4]. Over 400 million people suffer from G6PD deficiency worldwide, which is especially common in malaria-endemic regions [5]. In some parts of Myanmar, the prevalence of G6PD deficiency was 11.0%, according to a survey conducted in 1999–2003 [6]. By DNA analysis, people living in Shan State, Myanmar, had a high prevalence of G6PD deficiency, where 17.5% carried the G6PD Mahidol variant, with 11.8% in males and 21.0% in females [7]. Likewise, in Kachin State, G6PD deficiency was almost 20% [8]. To date, 185 G6PD single nucleotide polymorphism (SNP) sites have been described, with over 400 variants identified, most being single base changes [4]. These variants were mostly identified by biochemical analysis, based on the residual enzyme activity [2]. Males are hemizygous with only one X chromosome and can be either G6PD normal or deficient. However, a G6PD-deficient allele in females can be heterozygous and homozygous. Because of X chromosome inactivation, RBCs in a G6PD heterozygous female are mosaic, with one part G6PD-normal and the other G6PD-deficient, producing variable G6PD phenotypes. Generally, G6PD-heterozygous females exhibit less severe clinical symptoms than males [9].

G6PD deficiency can be diagnosed by qualitative, quantitative, and molecular methods. For a definitive diagnosis of G6PD deficiency, a quantitative analysis by a spectrophotometric assay is mandatory and recognized as the reference test [10]. However, this technique requires advanced laboratory settings and skillful technicians. In addition, the diagnosis of G6PD deficiency by spectrophotometry is poorly standardized and a definition of 100% activity needs to be established for each site and assayed again. Under field conditions, simple point-of-care tests that can qualitatively or quantitatively define the G6PD status of the patients, such as G6PD rapid diagnostic tests (RDTs) or biosensors, are desired. The CareStart^™^ G6PD RDT test showed good sensitivity and specificity when compared with the standard quantitative assay when diagnosing G6PD deficiency [11]. The CareStart™ G6PD Biosensor is a device that can measure the level of G6PD enzyme activity in U/dL. This hand-held device works like a glucometer and can identify heterozygous females with intermediate G6PD activity based on enzyme level. To obtain the appropriate G6PD enzyme activity in U/gHb, the G6PD activity obtained using the CareStart^™^ G6PD Biosensor requires further normalization with haemoglobin level. However, this device has not yet been validated with a haemoglobin reader in Myanmar.

Myanmar carries the heaviest malaria burden in the Greater Mekong Subregion (GMS) and plans to eliminate malaria by 2030 [12]. The predominance of *Plasmodium vivax* malaria presents a major challenge to malaria elimination. Primaquine mass drug administration (MDA) has been a strategy successfully deployed in vivax-endemic areas [13]. However, this strategy requires a good understanding of the G6PD deficiency status in the target region. A critical issue for malaria control in Myanmar is that of mobile and remote populations, such as military personnel, who are exposed to a higher risk of malaria infection. Therefore, this study aimed to determine the prevalence of G6PD deficiency among military populations and their families. This study also aimed to appraise the efficacy of both the qualitative CareStart™ G6PD RDT and the quantitative Biosensor, with a standard spectrophotometry assay used as a comparative measure. The prevalence of the common G6PD variants in the study population was also assessed using a molecular technique.

## Methods

### Population and sample collection

**Fig. 1:**
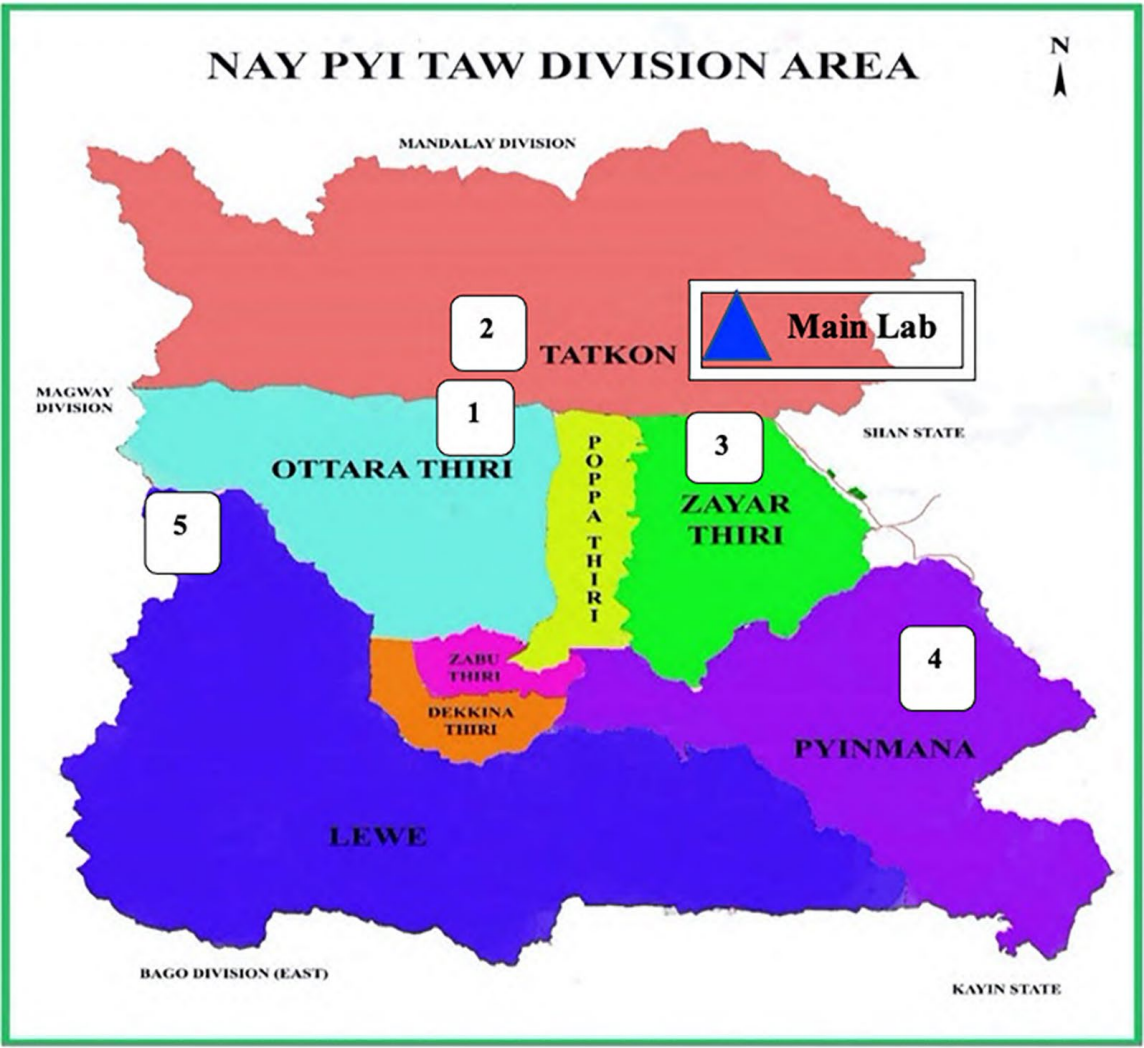
Location of the five study sites from Tatkon, Ottara Thiri, and Zayar Thiri Township, Nay Pyi Taw division area

A cross-sectional survey was conducted in Nay Pyi Taw, Myanmar. A total of 772 blood samples were collected from military personnel and their families residing in military camps in Tatkon (160 samples), Ottara Thiri (332 samples), and Zayar Thiri (280 samples) (Fig. 1). The Human Subjects protocol for this study was approved by the Ethical Review Committee of the Faculty of Tropical Medicine, Mahidol University, Thailand (MUTM2016-036-01), and the Institutional Review Board of the Defence Services Medical Research Centre (DSMRC), Myanmar (IRB/2016/60). Written informed consent was obtained before blood collection. Three millilitres of blood were obtained by venipuncture and collected in an EDTA tube. Blood was stored in a cooler box with ice packs during transportation from the sites to the DSMRC laboratory. Samples were processed for G6PD testing within 24 h after blood collection. An aliquot of the blood was spotted on Whatman 903 paper for later analysis of G6PD mutations.

### Determination of G6PD enzyme activity

The G6PD enzyme activity was determined using two quantitative assays (a spectrophotometry assay and a Biosensor assay). The qualitative G6PD test was performed with the CareStart^™^ G6PD RDT (Access Bio).

### Spectrophotometry

G6PD enzyme activity was determined by Randox Glucose-6-Phosphate Dehydrogenase-PD410 reagent kit (Randox Laboratories Ltd., UK) according to the manufacturer’s protocol with some modifications, and analysed by Microlab 300 semi-automatic biochemistry analyzer (Randox Monza, Ireland). Briefly, whole blood was centrifuged at 1000 x g for 10 min to remove plasma. Packed blood was washed 3 times with 2 ml of cold 0.9% NaCl solution to remove the buffy coat. One hundred microlitres of packed blood were mixed with 0.5 ml lysis buffer and incubated at 4 °C for 15 min. The sample was centrifuged at 1000 x g for 10 min to collect the haemolysate. The assay was performed by mixing 50 µl of haemolysate with the reaction solution (3 ml of R1 and 100 µl of R2 solution) with incubation at 37 °C for 5 min. Immediately after adding 50 µl of substrate solution (R3 solution), initial optical density (OD) was read at 340 nm at 37 °C, with three repeated measurements at 1 min intervals. Two control samples, normal and deficient (Randox), were analysed before running the test samples and repeated every 50 tests. The results were considered valid if the control values fell within the range provided by the company. Haemoglobin (Hb) concentration was determined using a haematology analyzer (ABX Micros ES 60, France). Enzyme activity (U/L) was adjusted by the haemoglobin value to generate the standard unit of G6PD activity (Unit per gram of Hb, U/g Hb).

### Biosensor

A CareStart^™^ G6PD analyzer (Access Bio, Korea) was used to quantify G6PD activity. After insertion of the test strip into the G6PD analyzer, the test strip window was filled with 10 µl of whole blood. Enzyme activity (U/dl) was adjusted by the Hb value obtained from the haematology analyzer to generate G6PD enzyme activity (U/g Hb).

### G6PD RDT

The CareStart^™^ G6PD RDT (Access Bio, Korea) was used to analyze G6PD activity qualitatively according to the manufacturer’s procedure. In brief, 10 µl of whole blood sample was added to the sample well, followed by two drops of assay buffer in the buffer well. The result was read in 10 min when a distinct purple colour appeared in the result window indicating normal G6PD, or very faint or no colour indicating G6PD deficiency.

### Determination of G6PD mutations by polymerase chain reaction-restriction fragment length polymorphism (PCR-RFLP)

DNA extraction was performed using the Chelex method according to the manufacturer’s procedure. Hb was released by incubating the blood filter with 1 ml of 0.5% saponin-PBS solution in a 1.5 ml microcentrifuge tube overnight at room temperature. After removing the brownish-red solution, the blood filter was washed once with 1 ml of 0.5% saponin-PBS solution. After discarding the supernatant, 200 µl of hot Chelex solution was added. The mixture was vortexed immediately and boiled for 5 min. The supernatant containing DNA was collected in a clean new tube.

The common G6PD mutations, including G6PD Mahidol, Viangchan, Chinese-4, Chinese-5, Kaiping, Canton, Union, and Mediterranean variants, were genotyped according to the established PCR-RFLP method [14]. In a 25 µl reaction, 10 µl of extracted DNA sample was mixed with 12.5 µl of GoTagGreen Master Mix and 1 µl of 10 µM forward and reverse primers. Amplification was performed on a Mastercycler nexus GSX1 (Eppendorf), with first denaturation at 95 ℃ for 5 min, followed by the PCR cycling conditions described in Additional file 1: Table S1. The final extension was performed at 72  ℃ for 10 min. Ten microlitres of the PCR product were digested at 37 ℃ with 10 units of appropriate restriction enzymes (New England Biolabs) in a 25 µl reaction for 2 h. The DNA bands were separated and visualized on 3% agarose gel.

### DNA sequencing

Direct sequencing of the PCR product was performed for G6PD-deficient samples whose G6PD mutations could not be identified using the PCR-RFLP procedure. The exon 2 to exon 13 fragments of the *G6PD* gene were amplified using the appropriate primer sets (Additional file 2: Table S2) [15, 16]. The amplicon was used as the template for DNA sequencing on a 3730xl DNA Analyzer (Thermo Fisher Scientific) using the same primer sets.

### Statistical analysis

The G6PD spectrophotometry assay was used as the reference assay. The adjusted median was calculated on males, as previously published [10]. The adjusted male median (AMM) was computed twice using both spectrophotometry and biosensor techniques. Subsequently, the samples were categories twice based on the AMM, and their performance was evaluated by comparing the resulting categories. The diagnostic performance (sensitivity, specificity, positive predictive value, negative predictive value, accuracy) of the G6PD biosensor at different cut-off values (30%, 70%, and 80%) was calculated. Bland-Altman plot and receiver operating characteristic curve were used to identify the appropriate enzyme activity cut-off for the CareStart^™^ G6PD biosensor. The enzyme activity cut-off from the CareStart^™^ G6PD biosensor assay corresponding to the 30% G6PD activity from the spectrometry assay was selected based on the Youden Index. The 30% G6PD activity from the standard spectrophotometry assay was used as a cut-off to identify G6PD deficiency. All statistical analyses were conducted in GraphPad Prism 9.0 (GraphPad Software, USA).

## Results

### Quantitative analysis of G6PD enzyme activity

**Fig. 2:**
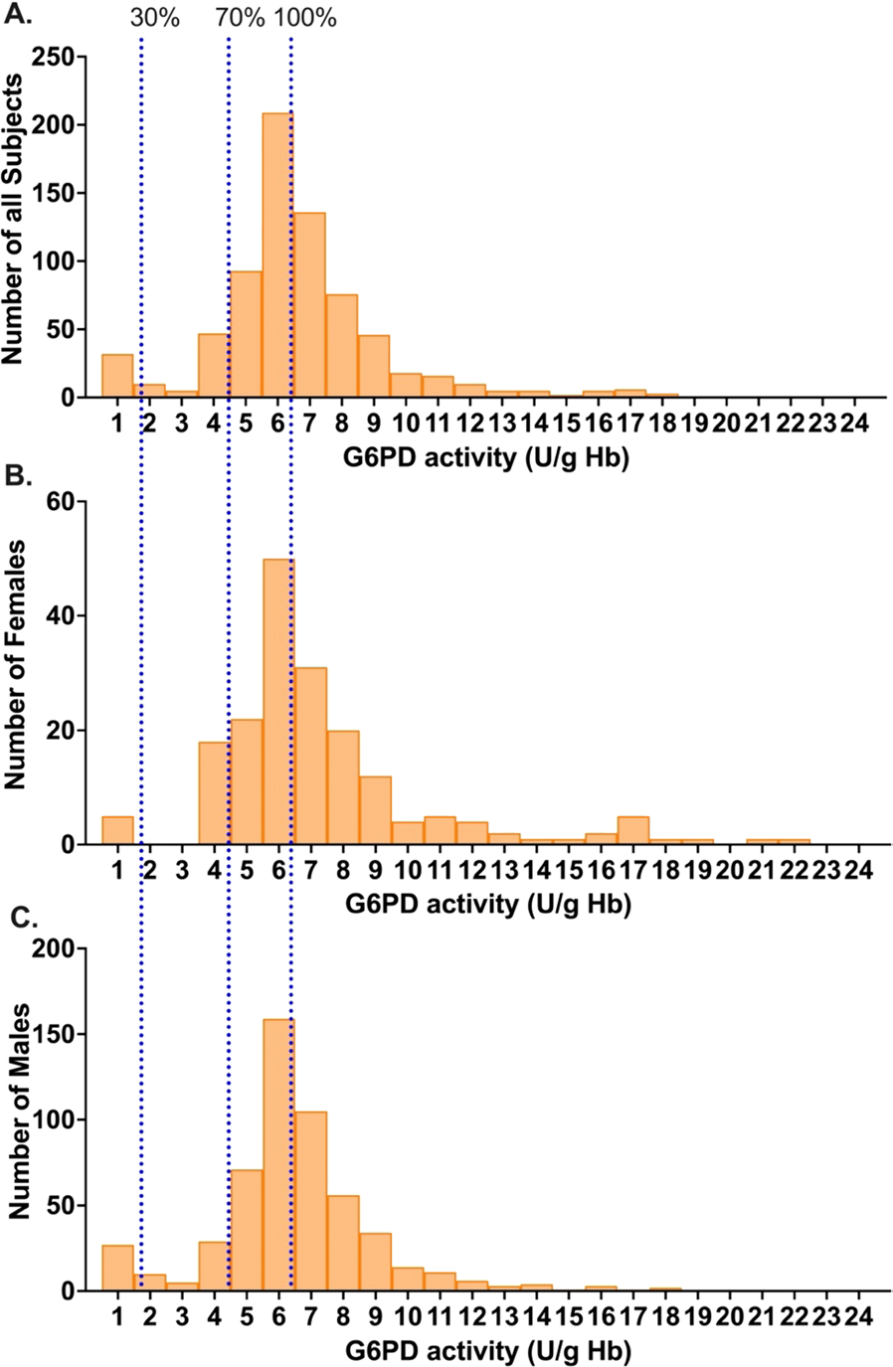
Distribution of G6PD activity (spectrophotometry and biosensor) among all samples. **A** Distribution of G6PD activity among all subjects, **B** distribution of G6PD activity among females, and **C** distribution of G6PD activity among all males. The adjusted male median (100% G6PD activity), 30%, and 70% reference G6PD activity are indicated in the blue dashed line

Quantitative measurement of G6PD activity using a standard spectrophotometry assay [17, 17] was performed on 772 blood samples collected from Tatkon, Ottara Thiri, and Zayar Thiri townships (579 males and 193 females). The majority of ethnic of participants were Myanmar (87.7%). Other ethnicities represented were Rakhine (3.8%), Kayin (3.8%), Shan (2.1%), Chin (1%), Kachin (0.6%), mixed (0.9%), and Mon (0.1%), respectively. To minimize the impact of severely deficient individuals on reference enzyme activity, the adjusted male median (AMM) was used to identify the reference value for the G6PD activity [10]. The overall AMM was determined to be 6.30 U/gHb and was used as the reference for 100% G6PD activity. The classical distribution of G6PD activity among females and males is shown in Fig. 2. The enzyme activity cut-off at 30% and 70% AMM was 1.89 U/g Hb and 4.41 U/g Hb, respectively. Based on the criteria for identification of deficient (< 30%), intermediate (30–<70%), and normal levels of G6PD activity (≥70%) [1], 85.9% (663/772) of the population had normal levels of G6PD activity (≥4.41 U/gHb). The intermediate G6PD activity level (1.89–4.41 U/g Hb) accounted for 3.2% of females. Even though the intermediate G6PD activity level was used mainly for females, 7.6% (44/579) of the male population also had G6PD levels in this category. The overall prevalence of G6PD deficiency in this population at a 30% cut-off (< 1.89 U/g Hb) was 10.9% (84/772). The prevalence of G6PD deficiency in males (12.4%, 72/579) was higher than in females (6.2%, 12/193) (Table 1).

**Table 1:** Prevalence of G6PD deficiency and reference value of G6PD enzyme activity in the study areas

Description	Over all Nay Pyi Taw	Ottara Thiri	Tatkon	Zayar Thiri
Total samples (males, females)	772 (579,193)	332 (299,33)	160 (80,80)	280 (200,80)
Age median (25,75 percentile)	32 (25,42)	33 (24,45)	32 (24,42)	30 (24,38)
History of malaria infection (%)	375/772 (49%)	190/332 (57%)	70/160 (44%)	115/280 (41%)
G6PD activity value (U/g Hb), SSA				
Adjusted male median (AMM)	6.3	6.6	7.8	5.8
80% of AMM	5.04	5.28	6.24	4.64
70% of AMM	4.41	4.62	5.46	4.06
30% of AMM	1.89	1.98	2.34	1.74
G6PD activity value (U/g Hb), CareStart^™^ G6PD biosensor				
Adjusted male median (AMM)	2.58	2.58		
80% of AMM	2.06	2.06		
70% of AMM	1.81	1.81		
30% of AMM	0.77	0.77		
Prevalence G6PD deficiency				
Spectrophotometry (< 1.89 U/g Hb)	10.9% (84/772)	12.0% (40/332)	13.1% (21/160)	8.2% (23/280)
CareStart^™^ G6PD RDT	10.5% (81/772)	11.7% (39/332)	11.3% (18/160)	8.6% (24/280)
CareStart^™^ G6PD biosensor (< 0.77 U/gHb)	12.67% (19/150)	12.67%(19/150)		

G6PD activity value by adjusted male median (80%, 70%, 30%) and prevalence of G6PD deficiency determined by Spectrophotometry and CareStart^™^ G6PD RDT in the study sites

**Fig. 3:**
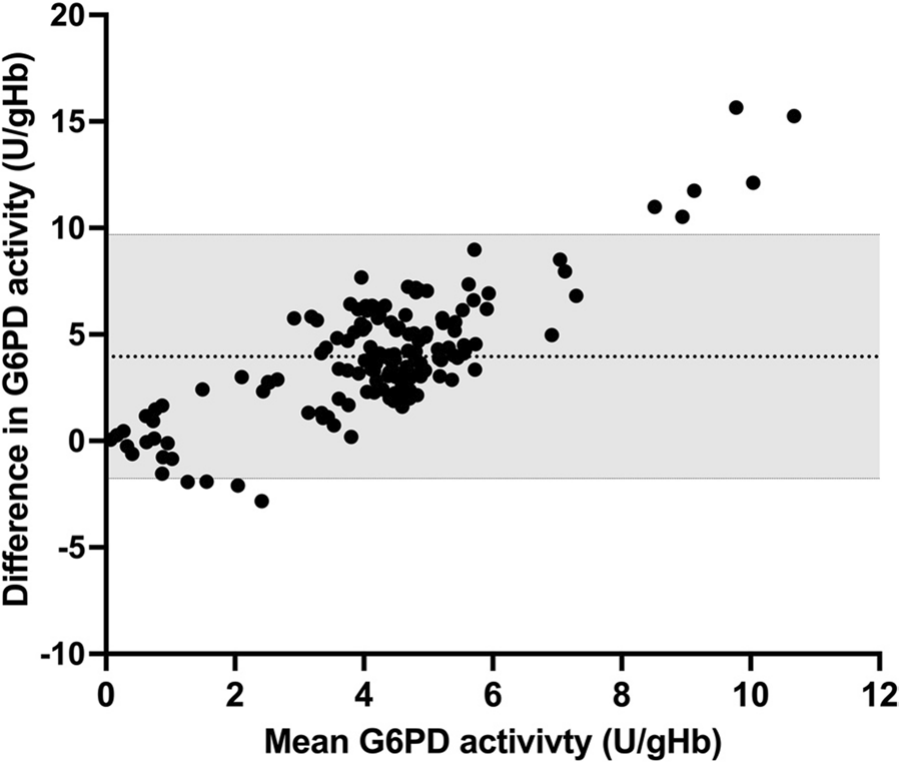
The bland-Altman plot of G6PD activity between CareStart^™^ G6PD biosensor and standard spectrophotometry assay. The dashed line represents the mean and the grey area represents the 95% limits of the agreement

**Fig. 4:**
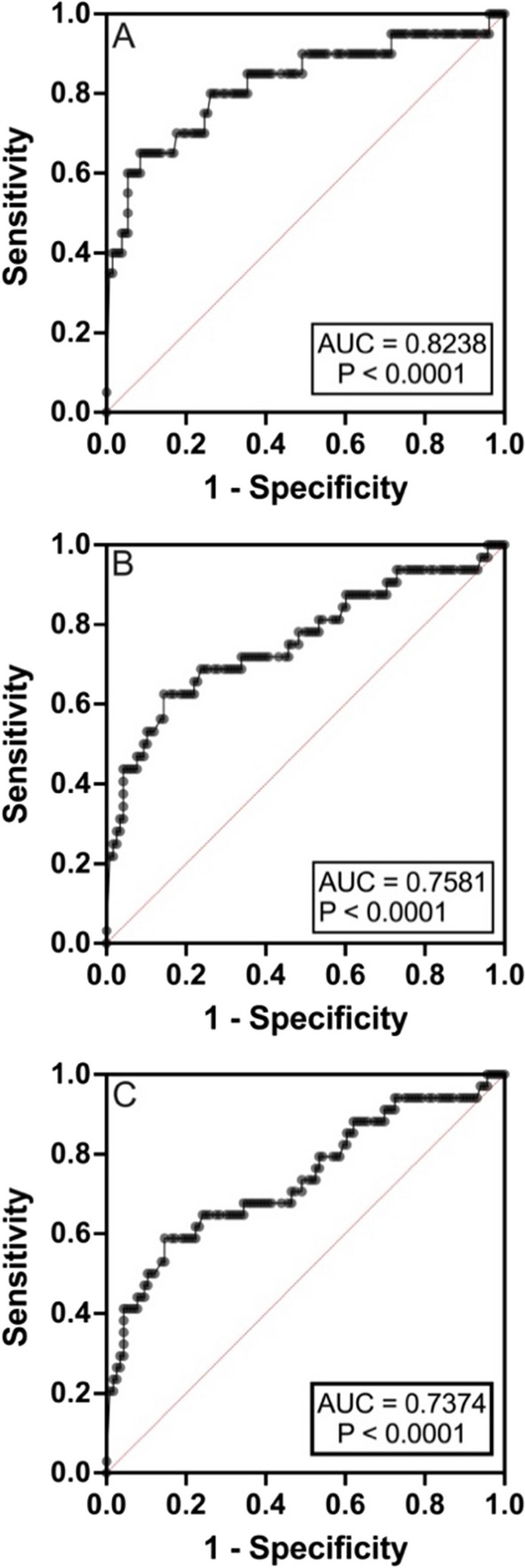
Receiver operator characteristic curve analysis and CareStart^™^ G6PD biosensor and SSA at 30% (Fig. 4A), 70% (Fig. 4B), and 80% (Fig. 4C) SSA-G6PD activity cut-off

The CareStart^™^ G6PD biosensor was used to assess the enzyme activity of 150 blood samples collected from Ottara Thiri. The Bland-Altman plot showed that the CareStart^™^ G6PD biosensor often underestimated G6PD enzyme activity, especially when the G6PD level was above the 30% cut-off (Fig. 3). The assay-specific AMM of the CareStart™ G6PD biosensor was 2.58 U/gHb. The diagnostic performance of the CareStart™ G6PD biosensor is shown in Table 2. The ROC analysis between the CareStart^™^ G6PD biosensor and spectrometry assays was evaluated at 30% (Fig. 4A), 70% (Fig. 4B), and 80% (Fig. 4C) of the G6PD activity cut-off from the spectrometry assay. The area under the curve (AUC) was 0.8238, 0.7581, and 0.7374 using the 30%, 70%, and 80% G6PD activity cut-offs, respectively. The performance of the CareStart^™^ G6PD in identifying G6PD deficiency at 30% assay-specific G6PD activity cut-off (0.77) U/g Hb is shown in Table 2 with 63% sensitivity, 95% specificity, 57% positive predictive value, 96% negative predictive value, and 92% accuracy. The prevalence of G6PD deficiency as defined by this assay was 12.67%.

**Table 2:** Diagnostic performance of CareStart^™^ G6PD biosensor at different cut-off points

CareStart^™^ G6PD Biosensor	30% G6PD activity cut-off (0.77 U/gHb) (95% CI)	70% G6PD activity cut-off (1.81 U/gHb) (95% CI)	80% G6PD activity cut-off (2.06 U/gHb) (95% CI)
Spectrophotometry cut-off	1.89 U/gHb	4.41 U/gHb	5.04 U/gHb
AUC (95%CI)	0.82	0.76	0.74
	(0.71–0.94)	(0.65–0.86)	(0.63–0.84)
Sensitivity	0.63 (0.38–0.84)	0.69 (0.50–0.84)	0.65 (0.47–0.80)
Specificity	0.95 (0.89–0.98)	0.75 (0.67–0.83)	0.70 (0.61–0.78)
PPV	0.57 (0.37–0.75)	0.24 (0.17–0.32)	0.19 (0.14–0.26)
NPV	0.96 (0.93–0.98)	0.96 (0.93–0.97)	0.95 (0.92–0.97)
Accuracy	0.92 (0.86–0.95)	0.75 (0.67–0.82)	0.69 (0.61–0.77)
Prevalence	10%	18%	20%
True positive	12/19	22/51	22/57
True negative	124/131	89/99	81/93
False positive	7/19	29/51	35/57
False negative	7/131	10/99	12/93

The performance of G6PD biosensor was calculated at 30%, 70% and 80% G6PD activity cut-off

### Qualitative analysis of G6PD activity

**Fig. 5:**
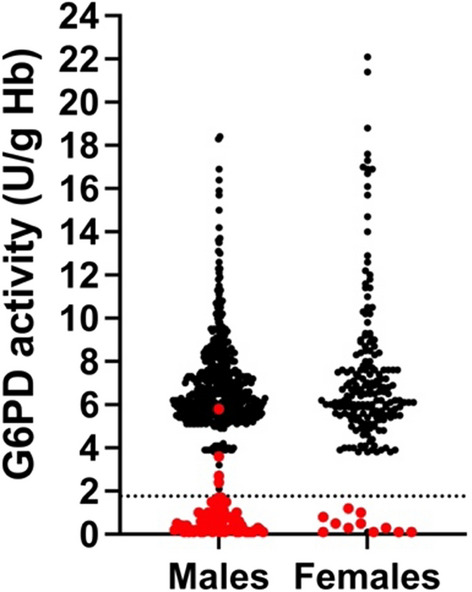
Distribution of G6PD enzymatic activity (UI/g Hb) values among males and females according to CareStart^™^ G6PD RDT test results. The red and black dots indicate G6PD deficiency and normal G6PD determined by RDT, respectively. 10% reference G6PD activity is indicated (horizontal black dash line)

The CareStart^™^ G6PD RDT was used to analyse all 772 samples. The overall prevalence of G6PD deficiency across the study sites was 10.5% (81/772), 12.1% (70/579) in males, and 5.7% (11/193) in females. The distribution of G6PD enzyme activity among G6PD-normal and G6PD-deficient individuals is shown in Fig. 5. G6PD deficiency was clearly separated from G6PD-normal at 30% G6PD enzyme activity. The diagnostic performance of the CareStart^™^ G6PD RDT compared with the spectrometry assay is shown in Table 3. The sensitivity and specificity were 93% and 98%, respectively. The CareStart^™^ G6PD RDT performed well in discriminating G6PD deficiency with 88% PPV and 99% NPV. Six males were G6PD-normal by the RDT but showed enzyme activity below the 30% cut-off.

**Table 3:** Diagnostic accuracy of G6PD RDT and G6PD biosensor

Parameters	G6PD RDT
Sensitivity (95% CI)	0.93 (0.85–0.97)
Specificity (95% CI)	0.98 (0.98–0.99
PPV (95% CI)	0.88 (0.79–0.94)
NPV (95% CI)	0.99 (0.98–0.99)
True positive	82/88
True negative	903/914
False positive	11/914
False negative	6/88

### G6PD genetic variants

G6PD genotyping was performed on all 193 females and 183 males, including all with G6PD deficiency (75 samples) defined by either spectrometry or RDT methods, and 108 G6PD-normal were selected randomly. G6PD mutations were found in 43.2% (79/183) of males and 19.2% (37/193) of females. The majority G6PD variant was the Mahidol variant (86.1% in males and 91.9% in females). 46% (46.0%; 47/102) of the samples with the Mahidol variant showed G6PD activity below the 10% cut-off value defined by the spectrometry method (< 0.63 U/g Hb), including one homozygous female (Table 4). The Andalus variant, not previously reported in Myanmar, was identified by sequencing *G6PD* from one hemizygous male.

**Table 4:** Distribution of residual G6PD enzyme activity and different mutations among male and female samples

G6PD variants	Residual enzyme activity				
	<10% (<0.6 U/g Hb)	10–30% (0.6–1.9 U/gHb)	30–70% (1.9–4.4 U/g Hb)	≥70% (≥4.4 U/g Hb)	Total
Males					
Mahidol	38	21	9	0	68
Chinese-4	4	0	2	0	6
Kaiping	1	2	1	0	4
Andalus	0	1	0	0	1
Females					
Mahidol (Heterozygous)	8	3	0	22	33
Mahidol (Homozygous)	1	0	0	0	1
Chinese-4	0	0	0	1	1
Union	0	0	0	2	2

## Discussion

In Myanmar, a 14 day course of low-dose primaquine with a blood schizonticide drug (Chloroquine, 800 mg per day for 3 days) is provided to *P. vivax* and *Plasmodium ovale* patients to clear hypnozoites without testing G6PD status, putting G6PD-deficient patients at risk of life-threatening haemolysis [18]. Quantitative spectrophotometry is the standard method for testing G6PD enzyme activity and the definitive diagnosis of G6PD deficiency [2]. However, no internationally accepted threshold currently exists to define it [19]. Therefore, in the absence of a local G6PD reference value, G6PD deficiency cannot be determined accurately. Although Myanmar has a high prevalence of G6PD deficiency [8], local evidence-based data on quantitative enzyme activity are lacking, and no proposed cut-off value for G6PD deficiency specific to the Myanmar population exists. Different studies of G6PD deficiency have used different reference cut-off values [20–22]. Using the Trinity Biotech spectrophotometric assay, Oo et al. reported a higher adjusted male median G6PD activity of 8.28 U/gHb among adults in Myanmar compared with the 6.3 U/gHb in this study [7]. The Trinity Biotech G6PD reagent was discontinued during the time of this study. The reference range obtained from each commercial kit only reflects the age range, geographical location, and characteristics of each studied population [17, 23]. Harmonization of the assay protocol and reagents being used for the determination of G6PD activity would help limit variability in the nationwide-reference range. The reference values proposed by this study only reflect the G6PD activity determined by Randox spectrophotometric assay. In the present study, the prevalence of G6PD deficiency (10.9%) was comparable to the study conducted in different areas of Myanmar (Yangon, Mandalay, Sagaing, Pyin-Oo-Lwin, Sittwe, Kawthaung) [24].

In efforts to eliminate malaria, *P. vivax* and *P. ovale* are the most challenging among the human malaria parasites due to their formation of hypnozoites in the liver causing relapse without new infection. The 8-aminoquinoline drugs, primaquine and tafenoquine, are effective in eliminating hypnozoites, but prior G6PD testing is recommended for a safe radical cure. In countries where tafenoquine, a newly FDA-approved 8-aminoquinoline drug, is prescribed, quantitative analysis of G6PD activity is mandatory as the drug is only provided to patients who have enzyme levels of at least 70%. In the context of resource-constrained settings, point-of-care quantitative G6PD analysis is highly beneficial, as it can be readily used at the same health-facility level where malaria cases are routinely managed, for prompt and better-informed treatment options. The diagnostic performance of the CareStart^™^ G6PD biosensor, which was evaluated in this study and also in other countries, varied with some improvement over time, but still requires further improvement to meet the acceptable target product profile [10, 17, 25–27]. The need for an additional haemoglobin analyzer to obtain haemoglobin values for normalizing G6PD activity also complicates the usage of the machine, especially in a field setting. The widely well-accepted STANDARD G6PD biosensor has been validated across multiple countries [28–30] and can, therefore, be implemented without establishing a laboratory or country-specific threshold. Field evaluations by health staff using the STANDARD G6PD biosensor at the patient’s first contact point have shown reliable results and can be used as an alternative to the standard spectrophotometry assay [29, 31].

The CareStart G6PD RDT is a qualitative test requiring no equipment. According to the recommendation of the World Health Organization (WHO), the qualitative G6PD rapid test should have more than 95% sensitivity compared with the standard spectrophotometric assays and > 95% NPV at the G6PD enzyme activity cut-off value of ≤ 30%. Besides, the preferred product should be stable at temperatures of 30–40 °C found in tropical countries and have a visual readout that clearly differentiates between “deficient” and “normal” patients [32]. The performance of the G6PD rapid test in this study was among the preferred qualitative products recommended by the WHO, but it had 93% sensitivity, which was slightly lower than the preferred 95% sensitivity. Unfortunately, the product was no longer available in the market. This also urges the need for a new G6PD diagnosis.

In Myanmar, there are eight main ethnicities with 135 sub-ethnic groups. The dominant G6PD deficiency mutation in Myanmar is G6PD-Mahidol, accounting for 91.3% of G6PD variants among various ethnic groups [6, 33]. Other variants have also been reported, including Kaiping, Viangchan, Union, Canton, Chinese-4, Chinese-5, Mediterranean, Acores, Seattle, Jammu, Coimbra, Kerala-Kalyan, and Valladolid [6, 34–36]. The results in this study were consistent with these earlier findings. The Chinese-5, Chinese-4, Kaiping, and Union mutations found in this study were common variants in the Chinese population [37], suggesting a past spread to Southeast Asian countries from China [38]. The Andalus variant, first described in 1990 [39] and previously reported in a Malaysian population [40], was identified in the Myanmar population for the first time. Notably, almost 60% of males with the Mahidol mutation in this study had < 10% of the normal G6PD activity, within the Class II severe deficiency range, which concurs with an earlier study on the Thailand-Myanmar border [35].

## Conclusion

Myanmar is a malaria-endemic country, and military personnel and people residing in remote border regions are at higher risk of infection. In Myanmar, G6PD testing is usually not done before administering primaquine, which carries a significant risk of clinically concerning haemolysis [41]. The quantitative or qualitative G6PD tests that are easy to use and at a cost comparable with the reference spectrophotometry assay or malaria RDT, respectively, should be deployed in remote endemic areas before radical treatment with primaquine or tafenoquine is prescribed. This study provided further information on the prevalence and variants of G6PD deficiency in the Myanmar population and evaluated the performance of the point-of-care test for the identification of G6PD deficiency.

## Supplementary Information

**Additional file 1: Table S1.** Primers and PCR-RFLP conditions for genotyping of G6PD variants. 

**Additional file 2: Table S2.** Primersset for amplification of different exons of G6PD encoding gene.

## Data Availability

The datasets used and/or analysed during the current study are available from the corresponding author upon reasonable request.

## Abbreviations

AMM: Adjusted male median
AUC: Area under the curve
DSMRC: Defense Sevices Medical Research Centre
G6PD: Glucose-6-phosphate dehydrogenase
Hb: Haemoglobin
NADPH: Nicotinamide adenine dinucleotide phosphate
NPV: Negative predictive value
OD: Optical density
PPV: Positive predictive value
PCR-RFLP: Polymerase chain reaction-restriction fragment length polymorphism
RDT: Rapid diagnostic test
ROC: Receiver operating characteristic [curve]
WHO: World Health Organization
