# Supplementary material for: Prevalence of G6PD deficiency and diagnostic accuracy of a G6PD point-of-care test among a population at risk of malaria in Myanmar
Source: Malar J. 2023 May 1;22:143. doi: 10.1186/s12936-023-04559-6 (PMC10150473; doi:10.1186/s12936-023-04559-6)
Supplement: Supplementary file 1 — Additional file 1: Table S1. Primers and PCR-RFLP conditions for genotyping of G6PD variants. [file 12936_2023_4559_MOESM1_ESM.docx]

**Table S1**. Primers and PCR-RFLP conditions for genotyping of G6PD variants

| **G6PD variants** | **Sequence (5’-3’)** | **PCR conditions** | **Restriction enzymes** | **Product size (bp)** |
| --- | --- | --- | --- | --- |
| Viangchan | F: TGGCTTTCTCTCAGGTCTAG | 35 cycles of 1 min denaturation at 95˚C, 1 min annealing at 54˚C, 1 min extension at 72˚C | Hind III | Wildtype=104 |
| 871 G>A | R: GTCGTCCAGGTACCCTTTGGGG |  |  | Mutant=82, 22 |
| Mahidol | F: GCGTCTGAATGATGCAGCTCTGAT | 35 cycles of 1 min denaturation at 95˚C, 1 min annealing at 55˚C, 1 min extension at 72˚C | Xba I | Wildtype =126 |
| 487 G>A | R: CTCCACGATGATGCGGTTCAAGC |  |  | Mutant=106, 20 |
| Chinese-5 | F: GTCAAGGTGTTGAAATGCATC |  | Mbo II | Wildtype=187 |
| 1024 C>T | R: CATCCCACCTCTCATTCTCC |  |  | Mutant=150, 37 |
| Chinese-4 | F: GGACTCAAAGAGAGGGGCTG |  | BstE II | Wildtype=188, 15 |
| 392 G>T | R: GAAGAGGCGGTTGGCCGGTGAC |  |  | Mutant=203 |
| Union | F: ACGTGAAGCTCCCTGACGC |  | Hha I | Wildtype=142, 45, 27 |
| 1360 C>T |  |  |  | Mutant=187, 27 |
| Kaiping |  |  | Nde I | Wildtype = 227 |
| 1388 G>A | R: GTGAAAATACGCCAGGCCTTA |  |  | Mutant =206, 21 |
| Canton |  |  | Afl II | Wildtype = 214 |
| 1376 G>T |  |  |  | Mutant = 194, 20 |
| Mediterranean | F: ACTCCCCAAGAGGGGTTCAAGG | 35 cycles of 1 min denaturation at 95˚C, 1 min annealing at 58˚C, 1 min extension at 72˚C | Mbo II | Wildtype = 377, 119 |
| 563 C>T | R: CCAGCCTCCAGGAGAGAGGAAG |  |  | Mutant = 277, 119, 100 |
