# Supplementary material for: Prevalence of G6PD deficiency and diagnostic accuracy of a G6PD point-of-care test among a population at risk of malaria in Myanmar
Source: Malar J. 2023 May 1;22:143. doi: 10.1186/s12936-023-04559-6 (PMC10150473; doi:10.1186/s12936-023-04559-6)
Supplement: Supplementary file 2 — Additional file 2: Table S2. Primersset for amplification of different exons of G6PD encoding gene. [file 12936_2023_4559_MOESM2_ESM.docx]

**Table S2**. Primers set for amplification of different exons of G6PD encoding gene.

| **Primers** | **Sequence (5’-3’)** |
| --- | --- |
| Exon 2 | F: CTCTAGAAAGGGGCTAACTTCTCAA |
|  | R: GGAATTCCTGGCTTTTAAGATTGGG |
| Exon 3-4 | F: AGGATGATGTAGTAGGTCG |
|  | R: CCGAAGTTGGCCATGCTGGG |
| Exon 5 | F: GTGTGTCTGTCTGTCGTGTC |
|  | R: CACGCTCATAGAGTGGTGGG |
| Exon 6 | F: GGGAGGGCGTCTGAATGA |
|  | R: ACCTTGGGCCTCTGTGGTG |
| Exon 7 | F: TCCACCTTGCCCCTCCCTGC |
|  | R: CCAGCCTCCCAGGAGAGAGG |
| Exon 8 | F: CATGCCCTTGAACCAGGTGA |
|  | R: GCATGCACACCCCAGCTC |
| Exon 9-10 | F: TTCTCTCCCTTGGCTTTCTC |
|  | R: TACAGAGAAGGAGCAGTGTG |
| Exon 11 | F: GAAGCCGGGCATGTTCTTCAAC |
|  | R: GTGAAAATACGCCAGGCCTTA |
| Exon 12 | F: ACGTGAAGCTCCCTGACGC |
|  | R: CCAGGGCTCAGAGCTTGTG |
| Exon 13 | F: TGCCTCTCCTCCACCCGTCA |
|  | R: GTCAATGGTCCCGGAGTC |
